# Time- and dose-dependent regulation of circular RNAs in the response of triple-negative breast cancer cells to ionizing radiation

**DOI:** 10.1007/s12094-026-04280-1

**Published:** 2026-02-26

**Authors:** Maria Papatsirou, Eleni Mavrogonatou, Dimitris Kletsas, Andreas Scorilas, Christos K. Kontos

**Affiliations:** 1 Department of Biochemistry and Molecular Biology, Faculty of Biology, National and Kapodistrian University of Athens, 15701 Athens, Greece; 2 Laboratory of Cell Proliferation and Ageing, Institute of Biosciences and Applications, National Centre for Scientific Research “Demokritos”, 15341 Athens, Greece; 3 Institute of Applied Molecular Biology – Biomarkers and Omics Technologies, University Center of Research and Innovation “Antonis Papadakis”, National and Kapodistrian University of Athens, Athens, Greece

**Keywords:** Radiotherapy, DNA damage response, P53 signaling, MicroRNAs (miRNAs), Gene expression, Cell cycle

## Abstract

**Background:**

Triple-negative breast cancer (TNBC) is the most aggressive breast cancer subtype, with few treatment options and an increased risk of radioresistance development. Circular RNAs (circRNAs) are non-coding molecules that have emerged as major regulators of cancer progression and therapy efficacy. In this study, we aimed to elucidate their role in radiation response mechanisms of TNBC cell lines.

**Methods:**

BT-20, MDA-MB-231, and MDA-MB-468 cells were exposed to two different doses of ionizing radiation and analyzed at multiple time points to elucidate the expression profiles of ten circRNAs with regulatory potential, namely circTP53, circFOXO3, circCCNB1, circABCC1, circFBXW7, circHIPK3, circNCOR1, circADAM9, circUBAP2, and circATAD2. These circRNAs were pre-amplified and quantified using quantitative real-time PCR with divergent primers, while p53 phosphorylation was assessed by western blot analysis. Next, bioinformatics analysis was performed to identify miRNA targets and enriched signaling pathways for selected circRNAs.

**Results:**

Ionizing radiation induced distinct temporal and dose-dependent circRNA expression patterns. Six circRNAs were characterized by progressive upregulation, peaking at 24 h, with circTP53 expression paralleling p53 phosphorylation patterns. Four circRNAs showed early suppression with limited recovery. Cell line-specific responses reflected TNBC molecular heterogeneity, with MDA-MB-231 cells displaying unique recovery patterns. In addition, functional analysis for circTP53, circFBXW7, and circATAD2 revealed enrichment in critical pathways including p53 signaling, FOXO regulation, MAPK signaling, and ubiquitin-mediated proteolysis.

**Conclusion:**

The cell line-specific radiation-dependent circRNA expression patterns reflect the molecular diversity of TNBC, and pathway enrichments indicate the potential applications of these circRNAs as radiation sensitivity biomarkers and therapeutic targets to improve TNBC treatment efficacy.

**Supplementary Information:**

The online version contains supplementary material available at 10.1007/s12094-026-04280-1.

## Introduction

Breast cancer (BC) is the most common global malignancy and the second leading cause of cancer death after lung cancer [1]. It is considered a highly heterogeneous disease and is classified into distinct subtypes: luminal A or B, human epidermal growth factor receptor-2 (HER2)-positive, and triple-negative breast cancer (TNBC) [2]. TNBC is defined by the lack of hormone receptors and HER2 amplification, and patients with this subtype show the shortest survival rates [3]. The primary treatment for TNBC patients involves surgery in conjunction with chemoradiotherapy. Radiation therapy is administered to around half of all cancer patients at some point during their illness, often as an adjuvant treatment [4]. While these strategies are effective for many cases of early-stage BC, recurrence and metastasis continue to be significant challenges that need to be overcome [5].

For the majority of patients undergoing breast-conserving surgery, as well as high-risk cases following mastectomy, radiotherapy serves as a key treatment option, offering clear clinical advantages, including a decreased risk of BC-related mortality [4, 6]. Ionizing radiation (IR) used in radiotherapy inhibits tumor growth primarily by inducing cell cycle arrest through multiple forms of DNA damage, particularly double-strand breaks (DSBs) [7]. DNA damage response (DDR) is crucial for repairing DSBs and preserving genomic stability, thereby protecting cells from apoptosis and malignant transformation [8]. The extent of apoptosis is closely linked to how cells respond to IR, and RNA expression can contribute to the epigenetic regulation of this process [9]. In particular, non-coding RNAs can influence whether a cell undergoes programmed cell death by regulating gene expression through chromatin remodeling, histone modifications, and DNA methylation, or as molecular sponges that sequester regulatory microRNAs (miRNAs) [10]. Moreover, non-coding RNAs are also intricately involved in regulating the cell cycle, inflammatory response, and epidermal growth factor receptor (EGFR)-related pathways after irradiation [11].

A type of non-coding RNA that has been in the spotlight in recent years is circular RNAs (circRNAs). circRNAs are single-stranded RNA molecules that lack 5΄–3΄ polarity [12, 13]. They are characterized by a covalently closed, continuous structure, rendering them resistant to degradation by exonucleases [13]. circRNAs are produced by back-splicing circularization of primary transcripts and serve a variety of functions mainly through miRNA sponging, such as transcription and alternative splicing regulation [14]. With an increasing body of research revealing the complex roles of circRNAs in cancer, their involvement in diverse regulatory networks has become clear. For example, circ_0025202 is downregulated in BC and has been linked to tamoxifen resistance [15]. Deregulated circRNAs are also found to participate in the development of resistance to IR by modulating pathways and cellular processes, including epithelial–mesenchymal transition, apoptosis, and autophagy [16]. In addition, circRNA expression was shown to undergo significant changes during cancer-related cell proliferation [16, 17]. This regulation is dynamic and responsive to cellular stressors like IR.

For these reasons, we hypothesized that IR exposure would induce time- and dose-dependent changes in circRNA expression profiles, which could influence treatment response in TNBC. So far, only a few mechanistic studies have established that individual circRNAs can modulate radiosensitivity in BC. However, in this study, we examined the expression dynamics of functionally relevant circRNAs that are implicated in DDR, cell cycle control, and transcription regulation in three well-characterized TNBC cell lines, providing a systems-level view of circRNA networks. Our study is the first to systematically map the dose-dependent and temporal dynamics of a targeted circRNA panel specifically in the context of TNBC radiation response, which were previously uncharacterized. Moreover, we present a new connection between circRNA regulation and the activation of DDR in this aggressive subtype by showing that circTP53 expression closely resembles p53 phosphorylation patterns.

## Materials and methods

### Propagation of TNBC cell lines

Three well-studied TNBC cell lines were used in this study: BT-20, MDA-MB-231, and MDA-MB-468. All cell lines were propagated in an incubator at 37 °C and adjusted CO_2_ concentration of 5% in DMEM high glucose culture medium supplied with *L*-glutamine [2 mM], penicillin/streptomycin (100 U/mL/100 μg/mL), and an adapted concentration of fetal bovine serum at 10% (v/v). Cells were subcultured at 80% confluency using a trypsin/EDTA (0.05%/0.02% w/v) solution in phosphate-buffered saline. All cell culture materials were purchased from Biowest (Nuaillé, France).

### Exposure to IR

To induce DNA damage, TNBC cells grown in 100 mm culture dishes were exposed to 4 or 10 Gy of γ-irradiation in a ^60^Co gamma source (Gamma Chamber 4000 A, Isotope Group, Bhadha Atomic Research Company, Trombay, Mumbai, India), at a rate of 2 Gy/min.

### RNA extraction and cDNA synthesis

Following homogenization, total RNA was extracted from all cell lines in three replicates at three time points: 2 h, 9 h, and 24 h post-exposure to the selected doses of IR, as well as from untreated cells (control samples at 0 h and 24 h). For this purpose, the TRIzol® Reagent (Ambion™, Austin, TX, USA) was used according to the manufacturer’s instructions. The RNA samples were diluted in DEPC-treated water and stored at − 80 °C until use. RNA concentration and purity were determined spectrophotometrically with the BioSpec-nano Micro-volume UV–Vis Spectrophotometer (Shimadzu, Kyoto, Japan), while sample integrity was verified by agarose gel electrophoresis.

Then, 1 μg of total RNA from each of the extracts was reverse transcribed using 100 U of the highly thermostable Maxima™ H Minus Reverse Transcriptase (Thermo Fisher Scientific Inc., Waltham, MA, USA) and random hexamer primers (New England Biolabs Ltd.), following the standard protocol.

### circRNA selection, pre-amplification, and quantification of selected circRNAs

To comprehensively cover the radiation-induced post-transcriptional regulation of the TNBC cell lines, we selected a panel of ten candidate circRNAs based on their established roles in key cellular pathways. In particular, we included circRNAs implicated in cell cycle regulation and DDR (circTP53, circFOXO3, circCCNB1), drug resistance- and survival pathways-associated circRNAs (circABCC1, circFBXW7, circHIPK3), and circRNAs involved in transcription regulation and cellular adaptation (circNCOR1, circADAM9, circUBAP2, circATAD2). This panel was designed to monitor temporal circRNA dynamics within complementary pathways crucial to TNBC radiosensitivity.

For the pre-amplification and quantification of each circRNA, two sets of divergent primers were designed (Table S1) to increase specificity. The reaction mixture of the first-round PCR contained 1 U of KAPA Taq DNA Polymerase (KAPA Biosystems Inc., Woburn, MA, USA), KAPA Taq Buffer A, 0.2 mM dNTPs, and divergent primers at a final concentration of 0.4 μM. PCR products were then diluted at a 1:100 ratio in nuclease-free H_2_O, followed by SYBR Green-based real-time quantitative PCR (qPCR) assays using 0.5 μL of the diluted products as a template and the inner, second pair of divergent primers for each amplicon (Fig. S1). The specificity of qPCR products was confirmed by assessing amplicon size through agarose gel electrophoresis (data not shown). circRNA expression levels were quantified using the comparative threshold cycle (Ct) method (2^−ΔΔCt^) [18, 19], with *GAPDH* as the internal reference gene (Fig. S2). All assays were conducted in triplicate, and the mean Ct value of the technical replicates was calculated prior to statistical analysis.

### Protein extraction

Protein extraction from all samples was performed by acetone precipitation, as it is a reliable and efficient method offering high recovery and ease of downstream processing. In brief, following the removal of the remaining aqueous phase overlying the interface, 60 μL of organic phase was mixed with 3 volumes (180 μL) of acetone. After brief vortexing, the mixture was incubated at 4 °C for 4 h to precipitate proteins, followed by centrifugation at 18,200×*g* for 10 min at 4 °C. The pellet was then resuspended in 200 μL SDS 1%, followed by incubation at 50 °C for 5 min. Protein concentration was measured using the Lowry protein assay [20].

### Western blot analysis

All protein samples were diluted at a 1:2 ratio in sample buffer containing 125 mM Tris–HCl, pH 6.8, 3% (w/v) SDS, 20% (v/v) glycerol, 125 mM β-mercaptoethanol, and 0.02% (w/v) bromophenol blue, as well as a mixture of protease and phosphatase inhibitors (Sigma, St. Louis, MO). Then, cell lysates were boiled at 100 °C for 2 min and clarified by centrifugation at 13,500 RPM for 2 min. SDS-PAGE was carried out in Bis–Tris polyacrylamide gels, and proteins were transferred to PVDF membranes (Perkin Elmer–Thermo Fisher Scientific, Waltham, MA, USA), as previously described [21, 22]. The antibody for phospho-p53 (Ser15) was obtained from Cell Signaling Technology (Hertfordshire, UK), while anti-α-tubulin as well as secondary horseradish peroxidase-conjugated goat anti-rabbit and anti-mouse antibodies were purchased from Sigma. For the detection of immune complexes, an enhanced ECL reagent kit (Merck Millipore, Darmstadt, Germany) was used.

### In silico functional analysis

Computational analysis was performed utilizing established bioinformatics platforms to identify putative miRNA binding sites within the sequences of circTP53, circFBXW7, and circATAD2 and elucidate their functional roles in TNBC radiation response. Potential miRNA-circRNA interactions were predicted through the miRDB database prediction algorithm, which enables systematic mapping of regulatory binding sequences [23]. Following the selection and classification of functionally important miRNAs, DIANA miRPath v.4.0 was utilized for downstream predicted and validated target identification [24]. Finally, WebGestalt was used for gene set enrichment analysis as well as pathway analysis to characterize the broader regulatory networks and cellular processes potentially governed by circRNA-mediated miRNA interactions [25]. Enrichment scores were considered significant when the *p* value was below 0.050.

## Results

### IR induces p53 phosphorylation in TNBC cell lines

**Fig. 1:**
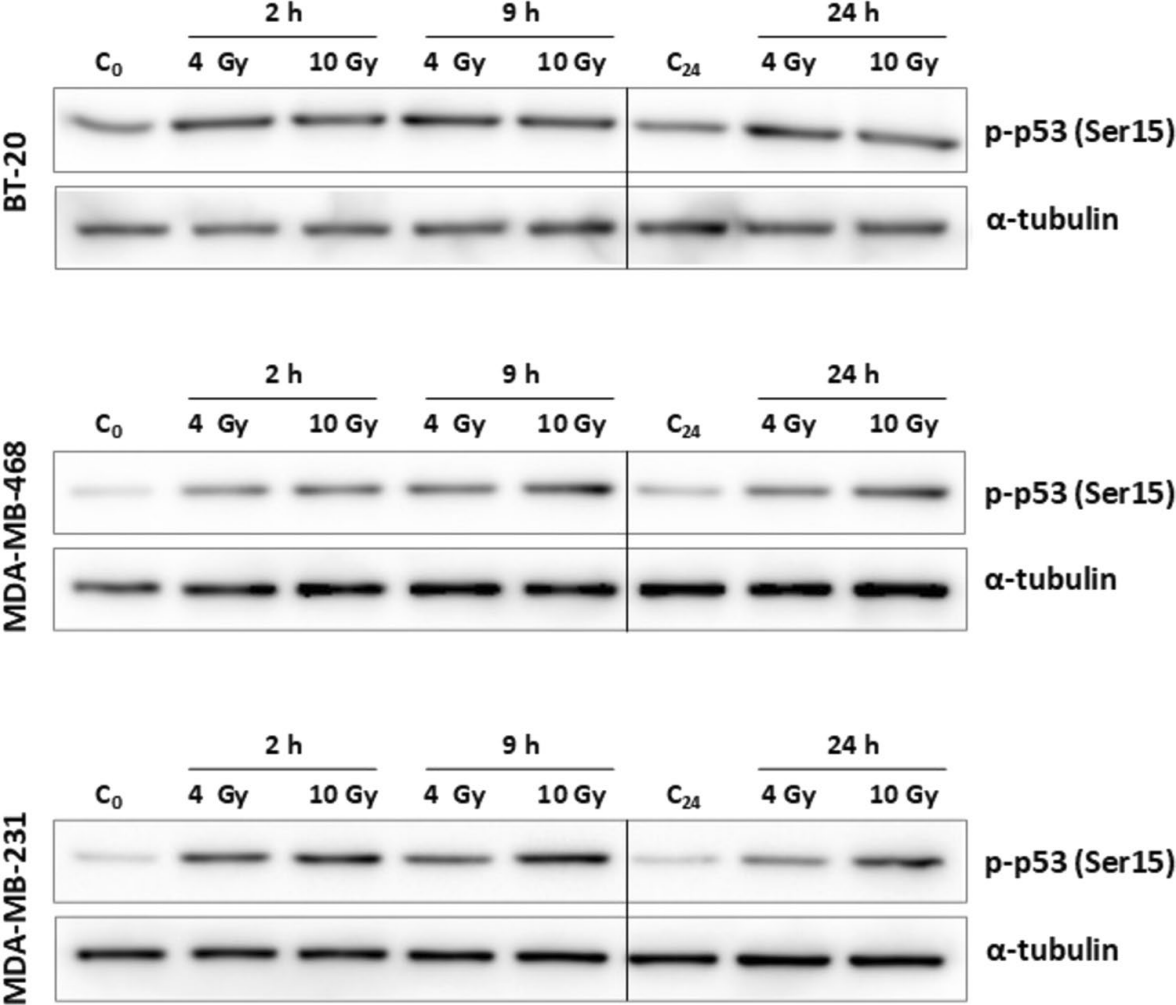
Western blot analysis of protein extracts from unirradiated and irradiated TNBC cells at different doses and time points for phospho-p53 (Ser15). The consistent α-tubulin expression across all samples validated equal protein loading. C = control, h = hours, Gy = gray

Western blot analysis revealed distinct patterns of time**-** and dose**-**dependent p53 phosphorylation at Ser15 post-exposure to IR across all TNBC cell lines (Fig. 1). Particularly, in BT-20 and MDA-MB-468 cells, p-p53 expression was markedly elevated at 2 h post-radiation at both 4 Gy and 10 Gy radiation doses, with sustained elevation at the 9-h time point. Notably, p-p53 levels remained elevated at 24 h in both dose groups compared to control conditions, indicating persistent DDR activation. MDA-MB-231 cells showed the most robust p-p53 response, with strong induction evident at 2 h that persisted through 24 h post-irradiation for both dose conditions. As expected, by 24 h, the p-p53 signal decreased in all three cell lines compared to the respective 24-h untreated controls, though it remained higher than that of the untreated controls.

### IR-responsive circRNAs show progressive upregulation with dose- and time-dependent kinetics

**Fig. 2:**
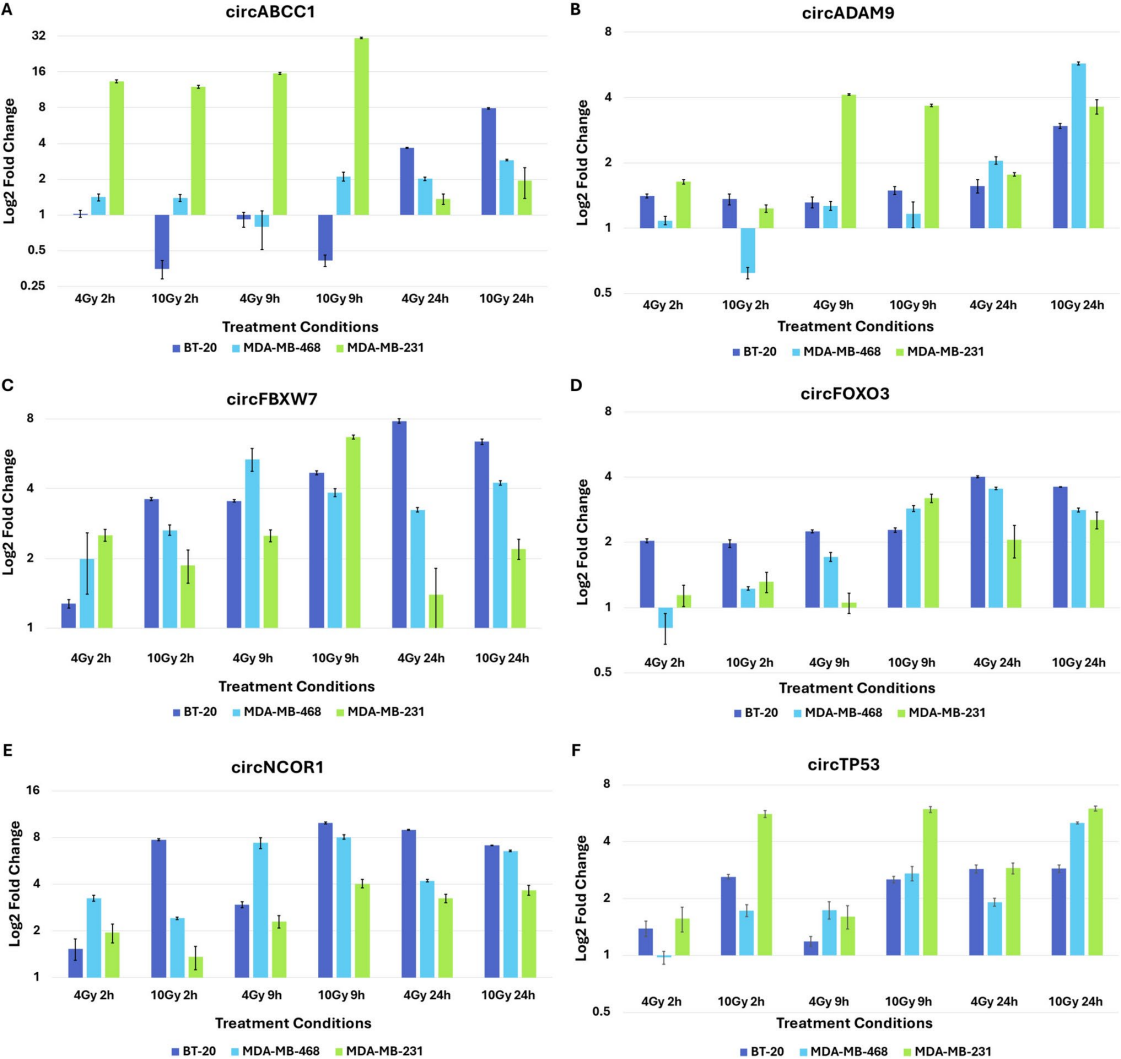
Expression levels of the radiation-induced circRNAs circABCC1 (**A**), circADAM9 (**B**), circFBXW7 (**C**), circFOXO3 (**D**), circNCOR1 (**E**), and circTP53 (**F**). These six circRNAs are consistently upregulated following irradiation, especially at higher doses and later time points. h = hours, Gy = gray

As demonstrated by qPCR analysis, six out of ten circRNAs exhibited upregulation after IR treatment. In particular, circABCC1 levels were significantly elevated in MDA-MB-231 cells at early time points (2–9 h post-radiation) and showed considerable regression at the 24-h time point, for both radiation doses (Fig. 2A). In contrast, in BT-20 and MDA-MB-468 cells, circABCC1 was notably upregulated at 24 h, with prior time points showing slight fluctuations in expression. circADAM9 displayed the highest expression levels at 24 h and 10 Gy for BT-20 and MDA-MB-468 cells, while in MDA-MB-231 cells, the 9-h time point showed the strongest induction for both radiation doses (Fig. 2B). circFBXW7 demonstrated progressive induction that peaked at later time points, particularly pronounced in BT-20 and MDA-MB-468 cells at 24 h; in MDA-MB-231 cells, the highest expression levels were reported 9 h post-radiation, especially for 10 Gy (Fig. 2C). circFOXO3 and circNCOR1 also displayed a gradual, dose- and time-dependent increase, with peak expression occurring at 24 h in both 4 Gy and 10 Gy treatment groups in all cell lines (Fig. 2D–E). Finally, circTP53 exhibited a clear dose-dependent increase that paralleled the p53 phosphorylation patterns observed in western blot analysis, with 10 Gy of radiation evidently being the most effective dosage for circTP53 induction, as exemplified in MDA-MB-231 cells (Fig. 2F).

### IR-repressed circRNAs show early suppression with limited recovery

**Fig. 3:**
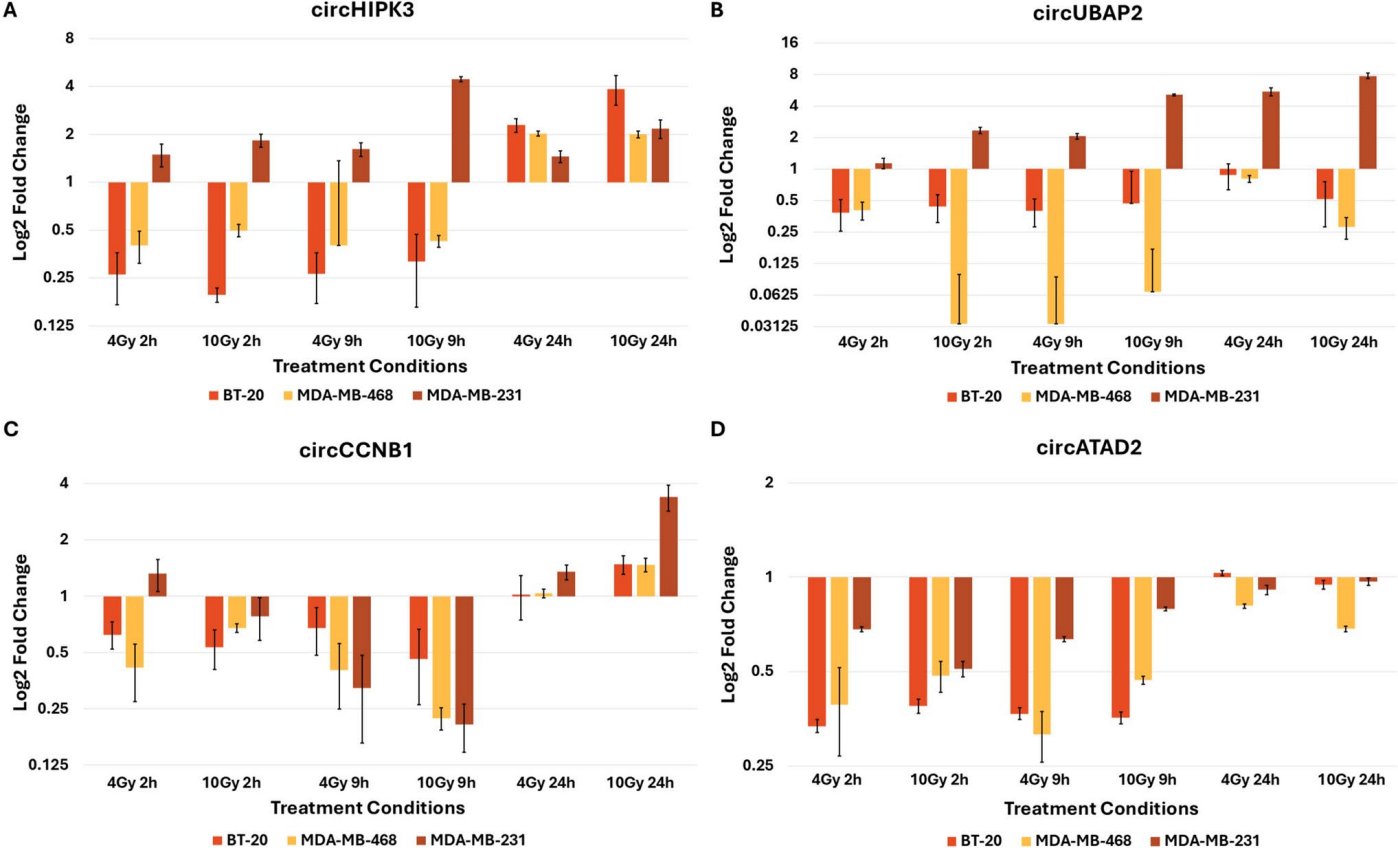
Expression levels of the radiation-suppressed circRNAs circHIPK3 (**A**), circUBAP2 (**B**), circCCNB1 (**C**), and circATAD2 (**D**). These four circRNAs show overall downregulation, particularly at early and intermediate time points, with only limited recovery later. h = hours; Gy = gray

Expression analysis revealed 4 circRNAs that are mainly downregulated following IR exposure, particularly at the early and intermediate time points. In detail, circHIPK3 and circUBAP2 levels were transiently reduced in BT-20 and MDA-MB-468 cells, with recovery of expression levels becoming apparent only at 24 h in some conditions (Fig. 3A, B). However, in MDA-MB-231 cells, both circRNAs were initially unaffected by radiation and then upregulated. In particular, circHIPK3 levels increased fourfold at 10 Gy/9 h, and circUBAP2 levels progressively increased at the highest dose of 10 Gy. circCCNB1 and circATAD2 exhibited consistent suppression across all experimental conditions (Fig. 3C, D). circCCNB1 clearly reached the lowest expression levels at 9 h, while both radiation doses had an immediate repressive effect on circATAD2. Minimal recovery was observed even at 24 h post-radiation in all three cell lines for both circRNAs. A notable exception of late induction was observed for circCCNB1 in MDA-MB-231 cells at 24 h (Fig. 3C).

### Functionally relevant miRNAs are predicted targets of circTP53, circFBXW7, and circATAD2

Three circRNAs of the above had distinguishable expression patterns in response to IR and were therefore chosen for downstream in silico functional analysis. More specifically, circTP53 and circFBXW7 exhibited a clear dose-dependent upregulation in all cell lines, while circATAD2 was uniformly downregulated across experimental conditions. According to our bioinformatic analysis, circTP53 possesses putative binding sites for 21 miRNAs, circFBXW7 for 25 miRNAs, and circATAD2 for 31 miRNAs. The miRNAs with the top ten prediction scores for each circRNA are shown in Table 1. Prediction scores ranged from 59 to 77 for circTP53, 71 to 92 for circFBXW7, and 76 to 96 for circATAD2, with circATAD2 showing the highest overall prediction scores. Most of the miRNA targets with the highest scores are well-studied cancer-related miRNAs that are implicated in major cellular processes and signaling pathways. In particular, the highest predicted binding affinity overall was found between circATAD2 and miR-5683, a known oncogenic miRNA in mutant p53-driven tumors, with a prediction score of 96. For circFBXW7, the top predicted target was miR-22-5p, a promising biomarker in BC, with a prediction score of 92. The highest-scoring miRNA predicted to bind to circTP53 was miR-1305, which is linked to androgen receptor signaling in TNBC, with a prediction score of 77.

**Table 1:** The putative target microRNAs (miRNAs) of circTP53, circFBXW7, and circATAD2 with the ten highest prediction scores^a^

circTP53		circFBXW7		circATAD2	
**Target miRNA**	**Prediction score**^a^	**Target miRNA**	**Prediction score**^a^	**Target miRNA**	**Prediction score**^a^
miR-1305	77	miR-22-5p	92	miR-5683	96
miR-4659a/b-3p	73	miR-6825-5p	89	miR-6504-3p	89
miR-466	72	miR-6072	82	miR-10399-5p	89
miR-4632-3p	71	miR-4677-5p	81	miR-6839-5p	86
miR-4643	69	miR-6891-3p	78	miR-449c-5p	83
miR-374a-3p	66	miR-6780a-3p	77	miR-34b-5p	83
miR-7160-5p	62	miR-4494	75	miR-4642	83
miR-3118	61	miR-6728-3p	75	miR-378j	83
miR-134-5p	61	miR-1226-3p	72	miR-2682-5p	78
miR-199b-5p	59	miR-502-5p	71	miR-10395-3p	76

^a^Calculated using the miRDB database prediction algorithm

### The regulatory effect of selected circRNAs is demonstrated in radiation response networks

**Fig. 4:**
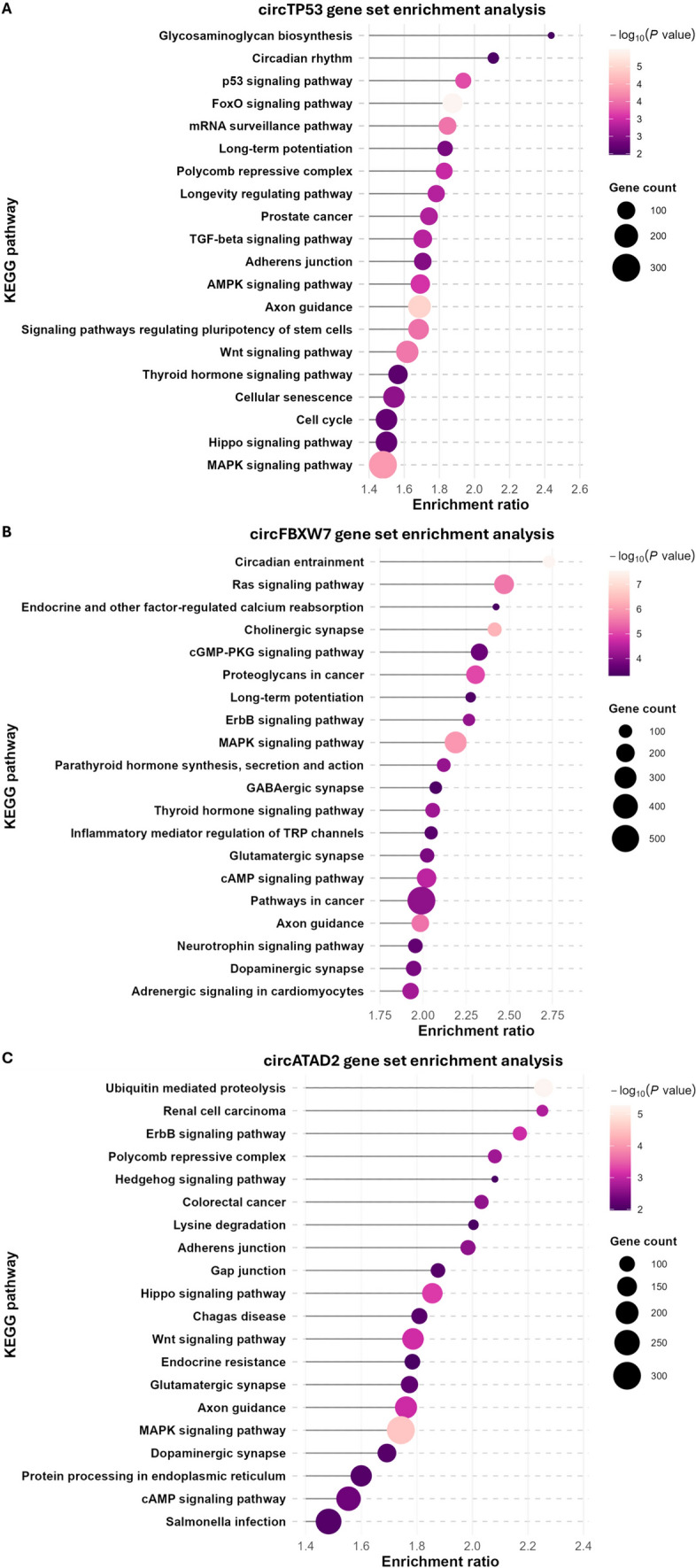
Significantly enriched KEGG pathways were revealed by in silico functional analysis, implicating circTP53 (**A**), circFBXW7 (**B**), and circATAD2 (**C**) in radiation response networks. The top 20 results are depicted for each circRNA. The size of each bubble shows the number of genes related to each pathway, while the color of each bubble represents the *P* value

Further analysis was conducted for the miRNA targets with the three highest prediction scores for each circRNA, regarding their putative and validated binding sites in mRNAs. This way, circTP53 had 4,001 unique target genes, circFBXW7 was associated with 2,527 unique genes, and circATAD2 had 2,003 unique downstream targets. Then, gene set enrichment analysis through the Kyoto Encyclopedia of Genes and Genomes (KEGG) functional database revealed enriched radiation response pathways in breast cancer, in which these circRNAs might participate. The results for circTP53 revealed significant enrichment for the p53, FOXO, and AMPK signaling pathways (Fig. 4A), which are greatly implicated in DDR, apoptosis regulation, and treatment resistance of breast cancer, as well as the mRNA surveillance pathway and the signaling pathways regulating pluripotency of stem cells, both critical systems in the context of radiation response. The Polycomb repressive complex pathway was also significantly enriched, further implicating circTP53 in DNA repair mechanisms as well as chromatin remodeling and epigenetic regulation. In addition, circFBXW7 seems to be intricately involved in the MAPK and Ras signaling pathways and the Proteoglycans in cancer pathway (Fig. 4B), which are core TNBC signaling and growth control axes that directly regulate cancer cell behavior. The cGMP-PKG signaling pathway, axon guidance, and cholinergic synapse pathways were also significantly enriched and could be relevant for understanding how cells coordinate responses to stress. Interestingly, the ubiquitin-mediated proteolysis was the most significantly enriched pathway for circATAD2 (Fig. 4C), highlighting its involvement in protein quality control and cellular survival responses in TNBC. Moreover, the MAPK, Hippo, Wnt, and ErbB signaling pathways also had remarkably high enrichment ratios, indicating the multifaceted role of circATAD2 in radiation treatment response of TNBC cells.

## Discussion

TNBC is the most clinically challenging subtype of breast cancer, defined by the lack of estrogen receptors, progesterone receptors, and HER2 amplification, thereby excluding the possibility of targeted hormonal or anti-HER2 treatments [26]. Because of its aggressive biological behavior, high potential for metastasis, and limited therapeutic options, TNBC is responsible for a disproportionate number of breast cancer-related deaths [3, 27]. Despite radiation therapy being the primary treatment option for TNBC patients, the heterogeneity of TNBC and its proclivity to develop radioresistance necessitate a better understanding of the molecular mechanisms that govern cellular responses to IR. The discovery of non-coding RNA networks that regulate radiation sensitivity may lead to the discovery of novel biomarkers for treatment stratification to improve radiation efficacy, which would ultimately improve the prognosis for TNBC patients [28, 29]. In this study, we thoroughly examined the dynamics of circRNA expression in TNBC cell lines in response to IR. Our approach identifies coordinated circRNA expression patterns that unfold from early to late post-radiation intervals, distinguishing conserved from cell line-specific signatures. These findings provide novel perspectives on the molecular mechanisms governing radiation response in TNBC, an important step toward establishing robust radiosensitivity biomarkers.

First, to investigate the cellular response to DNA damage, we sought to assess p53 phosphorylation status at serine 15 (p-p53). As evidenced by western blot analysis, IR treatment resulted in a significant and time-dependent increase in p-p53 protein levels in all three TNBC cell lines tested. In unstressed cells (control 0 h and 24 h), p-p53 expression was quite undetectable, confirming the tight regulation of p53 stability in unirradiated cells. These findings demonstrate that IR effectively activates the p53 DDR pathway, with distinct kinetic profiles that may reflect inherent differences in DNA repair capacity and stress response mechanisms [30]. Considering that p53 is mutated in BT-20, MDA-MB-468, and MDA-MB-231 cells, we hypothesized that regulatory circRNAs can either counteract the gain-of-function effects of mutant p53 or modulate radiosensitivity in a p53-independent manner.

The most notable finding was the upregulation of circABCC1, circADAM9, circFBXW7, circFOXO3, circNCOR1, and circTP53 following IR exposure. This coordinated response indicates that these circRNAs may work as part of a larger regulatory network rather than as individual molecular switches, by modulating critical determinants of post-radiation cell fate. The temporal kinetics observed, with peak circRNA expression occurring in most cases 24 h post-radiation, suggest that these circRNAs are involved in long-term adaptive responses rather than immediate damage-sensing mechanisms. This pattern corresponds to the established timeline of DDR, which includes initial damage detection and cell cycle checkpoint activation within hours, followed by extended DNA repair processes and cell fate decisions. Moreover, the dose-dependent circRNA upregulation that was prevalent in most cases, especially circTP53, circFOXO3, and circFBXW7, demonstrates that the regulatory networks in which these circRNAs are involved can respond proportionally to DNA damage. This dose–response relationship evidences that circRNA expression levels may serve as molecular indicators of the magnitude of cellular stress, potentially providing prognostic information about treatment efficacy.

Interestingly, the parallel between circTP53 upregulation and p53 phosphorylation patterns provides strong validation of our experimental system and suggests that circTP53 may function as a regulatory amplifier of p53-mediated responses. However, circTP53 is an established oncogenic circRNA in thyroid cancer and colorectal cancer, where it promotes tumorigenesis through miRNA sponging mechanisms [31, 32]. Interestingly, a recent study in head and neck squamous cell carcinoma further reinforces that circTP53 can engage the p53 regulatory machinery. It was shown that circTP53 interacts with USP10 and enhances USP10-dependent deubiquitination and stabilization of p53, with functional consequences that depend on p53 mutational status [33]. These findings highlight that circTP53 can modulate the p53 pathway output through protein stability control rather than only miRNA sponging. In parallel, recent literature on p53 signaling emphasized that the cellular response to IR is dictated by the integration of p53 dynamics with ubiquitination circuitry, transcriptional program selection, and context-specific rewiring of p53 functions [34–36]. The radiation-induced upregulation of circTP53 we observed may reflect the TNBC cells’ attempt to modulate the intensity and duration of the p53 pathway and the DDR signaling, which could either promote survival and repair or contribute to treatment resistance [37].

circFBXW7, circFOXO3, and circNCOR1 also appear to be key components of the universal DDR based on their robust sustained upregulation in all cell lines. All three circRNAs are well studied in the context of cancer progression and treatment resistance. In particular, circFOXO3 was recently found to be upregulated in glioma cells upon radiation exposure and to promote tumor radioresistance [38]. In parallel, circNCOR1 and circFBXW7 converge on regulatory pathways related to the cell cycle and ubiquitin-dependent processes. circNCOR1 has been shown to influence radiotherapy response via a CDK2-dependent mechanism, directly implicating it in S-phase progression and replication stress tolerance [39]. The parental gene of circFBXW7 is responsible for preventing unscheduled replication and maintaining genomic stability under stress; what is more, circFBXW7 is characterized as a tumor-suppressive circRNA that has been previously suggested to inhibit malignant progression and possess diagnostic potential in BC [40–42]. Moreover, circABCC1 is intricately linked to cell survival, since it supports post-IR survival by enhancing ROS handling and stress tolerance in the oxidative environment created by irradiation [43]. Finally, circADAM9 upregulation after IR is consistent with the activation of an adaptive module that can facilitate DNA repair capacity and survival, given that it has been shown to drive malignant phenotypes in BC [44, 45]. Our findings are in accordance with these studies and suggest that the radiation-induced circRNA upregulation may represent a beneficial cellular response that could enhance treatment efficacy.

On the other hand, circHIPK3, circUBAP2, circCCNB1, and circATAD2 underwent suppression following irradiation, providing interesting insights regarding cellular mechanisms that are employed as a response to genotoxic stress. In particular, circATAD2 has been found to promote the immune evasion of breast cancer cells [46], and it also stood out in our data as it was consistently downregulated across all experimental conditions. This finding suggests that suppression of chromatin remodeling activities may be a fundamental response to DNA damage. Given that the parental gene of circATAD2 has a key role in histone modifications and transcriptional regulation, it is possible that TNBC cells may temporarily limit chromatin remodeling activities to maintain genome stability during repair. In addition, the known requirement for cell cycle arrest following DNA damage aligns with the suppression of circCCNB1, which is involved in cell cycle regulation and DNA repair mechanisms [47, 48].

The cell line-specific recovery patterns that are prevalent in circCCNB1 and circATAD2, particularly the late induction observed in MDA-MB-231 cells, suggest that different cell lines may have varying capacities to resume proliferation after radiation-induced damage. This heterogeneity among cell lines is also evident for circABCC1, circFBXW7, circHIPK3, and circUBAP2. Overall, BT-20 and MDA-MB-468 cells demonstrated more maintained stress responses, while MDA-MB-231 cells showed unique responsiveness patterns, including early recovery of some circRNAs or contrasting circRNA expression profiles. This can be justified by the fact that BT-20 and MDA-MB-468 cells are considered more closely related regarding their transcriptomic similarities compared to MDA-MB-231 cells, which represent a distinct mesenchymal-like phenotype [49, 50]. The different genetic backgrounds and molecular subtypes within TNBC may be reflected in these cell line-specific variations, indicating that circRNA profiling may be used to stratify TNBC patients for individualized radiation therapy regimens.

The in silico functional analysis provided further mechanistic insights into how selected circRNAs may regulate radiation response and validated the biological relevance of our findings. Specifically, circTP53, circFBXW7, and circATAD2 may act as competing endogenous RNAs and regulate other RNA transcripts by competing for shared miRNA binding sites. These circRNAs are predicted to bind several functionally relevant miRNAs, which are known to inhibit the p53 pathway or its downstream effectors, as well as major signaling pathways that influence cell survival and apoptosis. In particular, miR-1305 inhibits the progression of various malignancies, such as non-small cell lung cancer, multiple myeloma, cervical cancer, and bladder cancer [51–54], while it can also regulate the p53-controlled cell cycle arrest programs through MDM2 [55]. In TNBC specifically, miR-1305 has emerged as part of a prognostic miRNA signature in patient cohorts [56]. Similarly, miR-22-5p is a known circulating miRNA and an early detection biomarker for BC, which is also identified among deregulated miRNAs in aggressive TNBC-associated regulatory axes, consistent with a role in post-transcriptional stress adaptation [57, 58]. Moreover, miR-5683 is implicated in the malignant proliferation of gastric cancer cells and can also exert antitumor effects through the downregulation of FGF2 within a mutant p53-associated regulatory pathway [59, 60]. These findings suggest that circRNAs have the capacity to intricately modulate survival signaling that is frequently engaged during recovery from DNA damage.

The enrichment of p53, FOXO, and AMPK signaling pathways in the circTP53 target gene network suggests that circTP53 may function as a central regulatory molecule coordinating multiple stress response pathways. In addition, the identification of the mRNA surveillance and pluripotency signaling pathway enrichment indicates that circTP53 may influence both quality control mechanisms and stem cell-like properties that are critical for long-term cellular survival. The relationship between growth factor signaling cascades and the DDR is highlighted by the enrichment of MAPK and Ras signaling pathways in the circFBXW7 network; circFBXW7 might act as a molecular link between internal damage response systems and external growth signals, potentially influencing treatment sensitivity. What is more, the predominant enrichment of the ubiquitin-mediated proteolysis pathway for circATAD2 indicates that this circRNA may undergo suppression in TNBC cells to downregulate protein degradation pathways to conserve resources during DNA repair processes.

It is important to recognize a number of limitations when interpreting these findings. First, the established cell lines used in this study might not accurately represent the complexity and diversity of primary TNBC tumors. The elements of the tumor microenvironment that could affect circRNA expression and radiation response in clinical settings are absent from the cell culture environment. In addition, three time points over 24 h were used for this analysis, which might not fully capture the dynamics of circRNA regulation, especially longer-term adaptive responses that might be important for clinical outcomes. Finally, computational prediction techniques were used for the functional analysis; these techniques need experimental validation of the predicted downstream effects and miRNA interactions.

However, future directions of this work include validation studies using primary TNBC patient samples, which would provide crucial evidence for the clinical relevance of the identified circRNA patterns. Correlation of circRNA expression levels with clinical outcomes, including local recurrence rates and radiation toxicity, would establish their prognostic and predictive value. In a clinical setting, the most plausible application is as a validated predictive circRNA panel to guide the choice between radiotherapy dose escalation and de-escalation. For example, TNBC patients exhibiting circRNA signatures associated with effective DDR activation might be candidates for treatment de-escalation approaches to minimize toxicity, such as altered fractionation schemes or combination with radiosensitizers or immunotherapy. Considering the inherent heterogeneity of TNBC, the incorporation of circRNA-based biomarkers into current molecular stratification systems may enhance personalized radiotherapy decision-making.

In conclusion, the development of circRNA-targeted therapeutic approaches is a promising area of research that is gaining traction among scientists. This study addresses a critical knowledge gap in understanding how circRNA networks coordinate cellular responses to genotoxic stress in the most aggressive breast cancer subtype, where treatment options are limited and radioresistance is a major clinical challenge. Integration of circRNA expression data with other omics approaches, including genomics, proteomics, and metabolomics, would provide a more comprehensive understanding of the molecular networks governing radiation sensitivity and resistance in TNBC cells.

## Supplementary Information

Below is the link to the electronic supplementary material.

Supplementary file1 (DOCX 928 KB)

Supplementary file2 (DOCX 870 KB)

Supplementary file3 (DOCX 24 KB)

## Data Availability

All data produced in the current study are available from the corresponding author upon reasonable request.
