# Supplementary material for: Time- and dose-dependent regulation of circular RNAs in the response of triple-negative breast cancer cells to ionizing radiation
Source: Clin Transl Oncol. 2026 Feb 26;28(8):3180–92. doi: 10.1007/s12094-026-04280-1 (PMC13401551; doi:10.1007/s12094-026-04280-1)
Supplement: Supplementary file 1 — Supplementary file1 (DOCX 928 KB) [file 12094_2026_4280_MOESM1_ESM.docx]

**
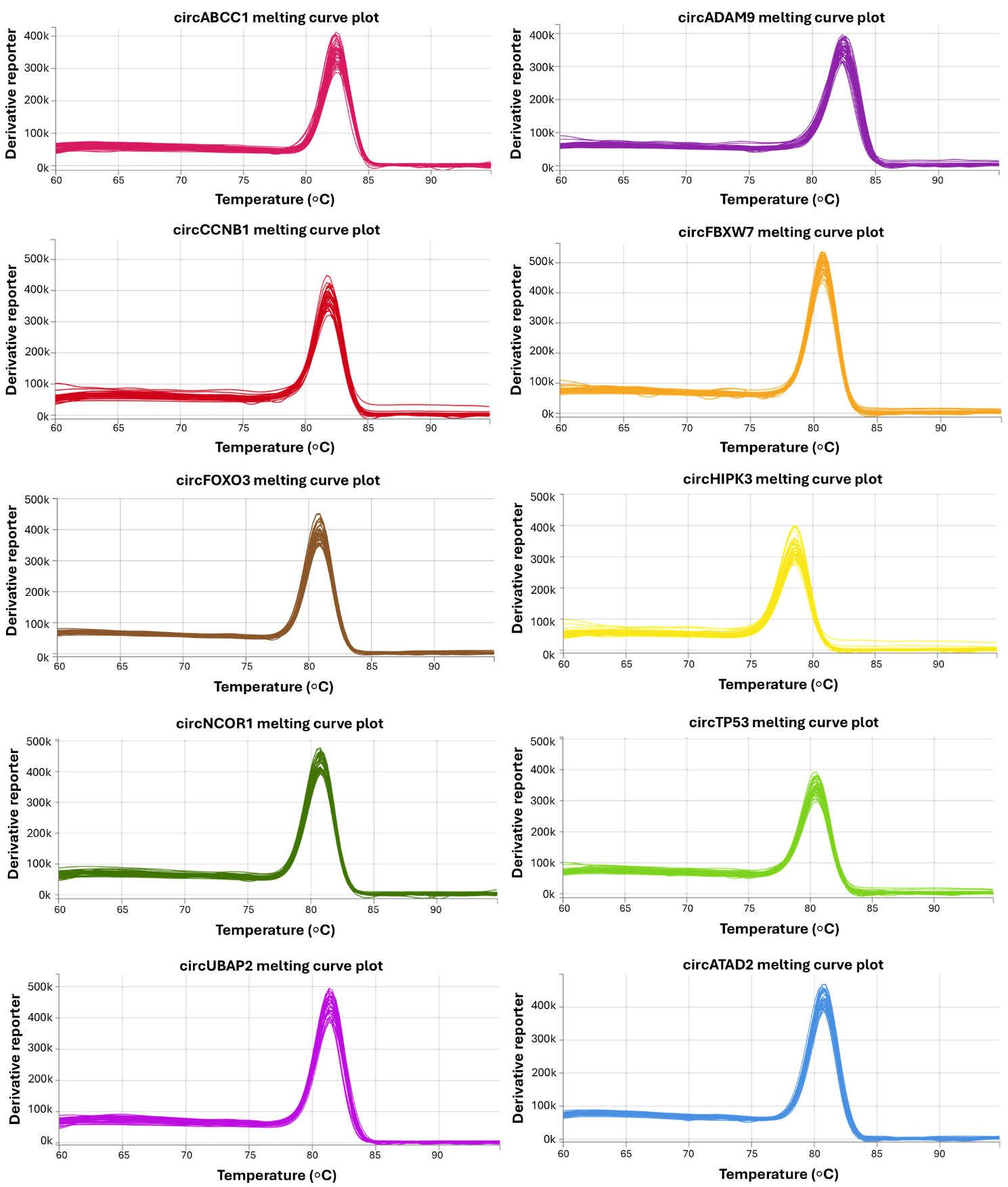
**

**Fig. S1** Melting curves of the 10 selected circRNA amplicons, produced after pre-amplification, demonstrate the unique amplification of each qPCR product. The approach of nested qPCR with divergent primers is designed to specifically amplify and quantify the selected circRNAs, ensuring the high specificity of the assay. Each plot shows the derivative reporter signal versus temperature. All assays demonstrate a single, sharp peak at the expected melting temperature, indicating specific amplification without detectable primer-dimer formation or non-specific products
