# Supplementary material for: Time- and dose-dependent regulation of circular RNAs in the response of triple-negative breast cancer cells to ionizing radiation
Source: Clin Transl Oncol. 2026 Feb 26;28(8):3180–92. doi: 10.1007/s12094-026-04280-1 (PMC13401551; doi:10.1007/s12094-026-04280-1)
Supplement: Supplementary file 2 — Supplementary file2 (DOCX 870 KB) [file 12094_2026_4280_MOESM2_ESM.docx]

**
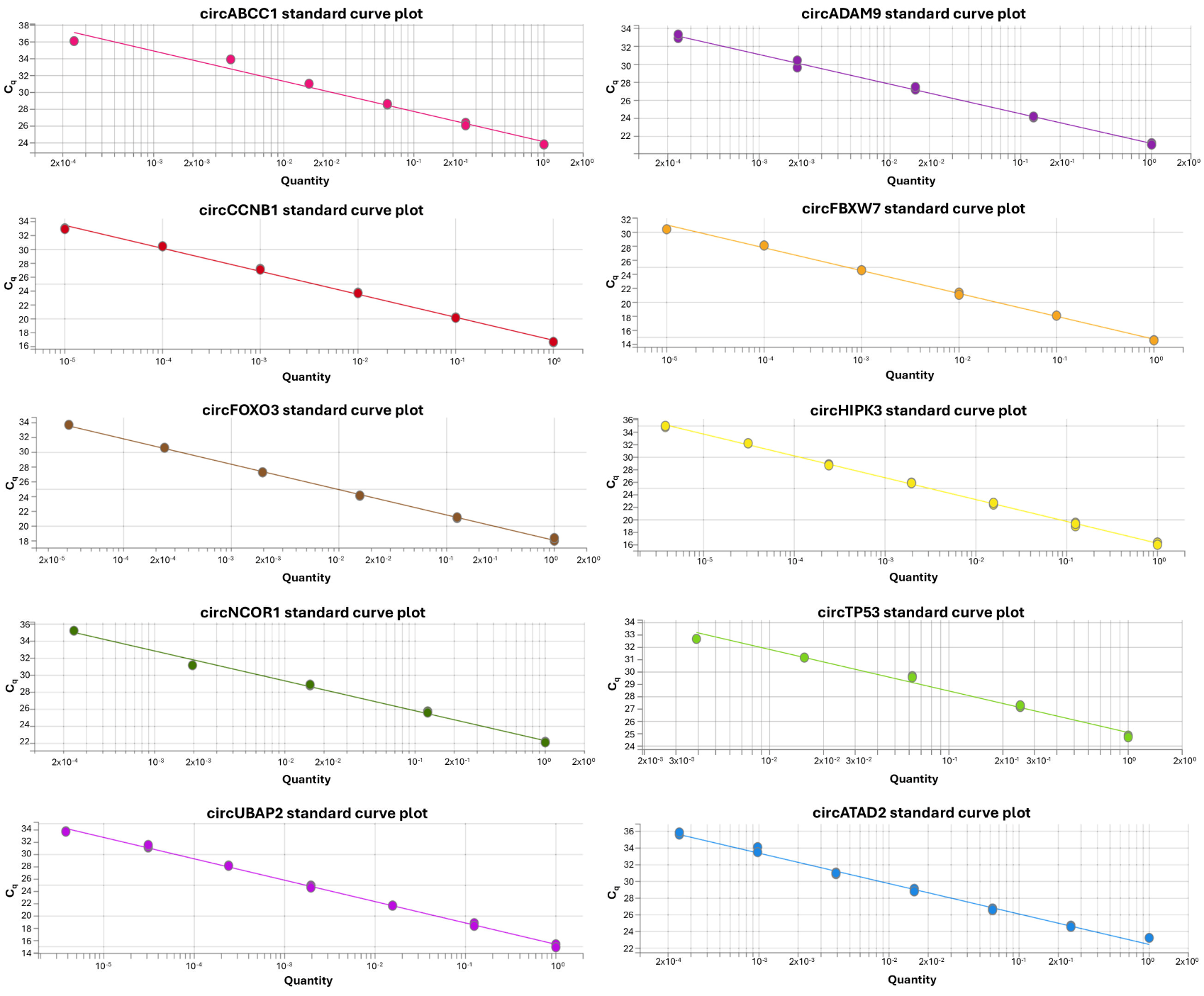
**

**Fig. S2** Standard curve plots for the 10 selected circRNA amplicons. Serial dilutions of cDNA were used to generate standard curves by plotting Ct values against the logarithm of template quantity. All primer sets exhibited strong linear correlation across the dilution range, and all amplicons were produced and amplified with high reaction efficiency (90-100%), as indicated by the slope of the corresponding standard curves
