# Supplementary material for: Time- and dose-dependent regulation of circular RNAs in the response of triple-negative breast cancer cells to ionizing radiation
Source: Clin Transl Oncol. 2026 Feb 26;28(8):3180–92. doi: 10.1007/s12094-026-04280-1 (PMC13401551; doi:10.1007/s12094-026-04280-1)
Supplement: Supplementary file 3 — Supplementary file3 (DOCX 24 KB) [file 12094_2026_4280_MOESM3_ESM.docx]

**Table S1** Primer sets utilized for pre-amplification and nested or semi-nested quantitative PCR (qPCR) assays of the selected circRNAs and *GAPDH*

| **Target** | **Assay** | **Primer direction** | **Primer sequence (5′ 🡪 3′)** | **T_a_ (°C)** |
| --- | --- | --- | --- | --- |
| *GAPDH* | Pre-amplification | Sense | CCACATCGCTCAGACACCAT | 60 |
|  |  | Antisense | TGACAAGCTTCCCGTTCTCA |  |
|  | Nested qPCR | Sense | ATGGGGAAGGTGAAGGTCG | 60 |
|  |  | Antisense | GGGTCATTGATGGCAACAATATC |  |
| circABCC1 | Pre-amplification | Sense | ACCATGCTGCTTGCTACCTT | 61 |
|  |  | Antisense | AAAATCCCAAGGCAGGGTCGT |  |
|  | Nested qPCR | Sense | TTCTGGCTGGTAGCCCTAGT | 60 |
|  |  | Antisense | GTCACGAAACAGGTCCACCT |  |
| circADAM9 | Pre-amplification | Sense | GTTGGACTGGAGATTTGGACC | 60 |
|  |  | Antisense | CCCATCATGTCATAAGAACTAGCTG |  |
|  | Nested qPCR | Sense | GTTGGACTGGAGATTTGGACC | 60 |
|  |  | Antisense | CACGAAGTTCCCCAGCACAT |  |
| circFBXW7 | Pre-amplification | Sense | AGCACACTGCAAGGAATG | 55 |
|  |  | Antisense | CCTAAGGAAGTAATCTTTTGTTG |  |
|  | Nested qPCR | Sense | CCCAGCAAGGACAGTTGGAA | 60 |
|  |  | Antisense | GAACGGGCAGGTCCACAATA |  |
| circFOXO3 | Pre-amplification | Sense | TCTCTGAACTCCCTACGCCA | 60 |
|  |  | Antisense | GGATGGAGTTCCTGCTTTGC |  |
|  | Nested qPCR | Sense | AGAAGTTCCCCAGCGACTTG | 60 |
|  |  | Antisense | TCCCCACGTTCAAACCAACA |  |
| circNCOR1 | Pre-amplification | Sense | GACCCAGGTGAATGACAGCA | 60 |
|  |  | Antisense | TTTCTGCACCTCTGCCTCTC |  |
|  | Nested qPCR | Sense | AGCTGACTCTGTGGACGTTG | 60 |
|  |  | Antisense | TCGGGCCTTTGGGCATTTAT |  |
| circTP53 | Pre-amplification | Sense | GAACAGCTTTGAGGTGCGTG | 60 |
|  |  | Antisense | CAGGGGAGTACTGGAGTGAG |  |
|  | Nested qPCR | Sense | CCTCTCCCCAGCCAAAGAAG | 60 |
|  |  | Antisense | TCTCGGAACATCTCGAAGCG |  |
| circCCNB1 | Pre-amplification | Sense | GGTTGGTGTCACTGCCATGT | 60 |
|  |  | Antisense | GCACACAATTATTCCATTCACCAT |  |
|  | Nested qPCR | Sense | CGGCCTCTACCTTTGCACTT | 60 |
|  |  | Antisense | AGGAGGAAAGTGCACCATGT |  |
| circHIPK3 | Pre-amplification | Sense | TTCTTGGTCGAGGCACGTTT | 61 |
|  |  | Antisense | TGAGGCCATACCTGTAGTACCG |  |
|  | Nested qPCR | Sense | GTGGCCACTGCACTGAAAAA | 60 |
|  |  | Antisense | TAAGGCTGCCGAACAGGATC |  |
| circUBAP2 | Pre-amplification | Sense | GCACTTCTGCCGTCCACATC | 61 |
|  |  | Antisense | TCACAGACGGGAGGGCA |  |
|  | Nested qPCR | Sense | CTCAGCTTAGCAGTTCGCTCT | 60 |
|  |  | Antisense | TGTTGTCTTTGGAGTGTGTGTGAC |  |
| circATAD2 | Pre-amplification | Sense | AGACTTTGAGCAGCTCTGTGA | 59 |
|  |  | Antisense | AGCACAGGCTCTATGCCTAA |  |
|  | Nested qPCR | Sense | TCCTCCAAATATGCCCCGTC | 60 |
|  |  | Antisense | AATCAGGAACCAGGAGTGCT |  |

T_a_: primer annealing temperature.
